# The Impact of Different Environmental Conditions on Cognitive Function: A Focused Review

**DOI:** 10.3389/fphys.2015.00372

**Published:** 2016-01-06

**Authors:** Lee Taylor, Samuel L. Watkins, Hannah Marshall, Ben J. Dascombe, Josh Foster

**Affiliations:** ^1^ASPETAR, Qatar Orthopaedic and Sports Medicine Hospital, Athlete Health and Performance Research CentreDoha, Qatar; ^2^Applied Sport and Exercise Physiology Research Group, Department of Sport Science and Physical Activity, Institute for Sport and Physical Activity Research, University of BedfordshireBedford, UK; ^3^Applied Sport Science and Exercise Testing Laboratory, Faculty of Science and Information Technology, University of NewcastleOurimbah, NSW, Australia

**Keywords:** cognitive function, cognition, heat, cold, altitude, hypoxia, environment

## Abstract

Cognitive function defines performance in objective tasks that require conscious mental effort. Extreme environments, namely heat, hypoxia, and cold can all alter human cognitive function due to a variety of psychological and/or biological processes. The aims of this Focused Review were to discuss; (1) the current state of knowledge on the effects of heat, hypoxic and cold stress on cognitive function, (2) the potential mechanisms underpinning these alterations, and (3) plausible interventions that may maintain cognitive function upon exposure to each of these environmental stressors. The available evidence suggests that the effects of heat, hypoxia, and cold stress on cognitive function are both task and severity dependent. Complex tasks are particularly vulnerable to extreme heat stress, whereas both simple and complex task performance appear to be vulnerable at even at moderate altitudes. Cold stress also appears to negatively impact both simple and complex task performance, however, the research in this area is sparse in comparison to heat and hypoxia. In summary, this focused review provides updated knowledge regarding the effects of extreme environmental stressors on cognitive function and their biological underpinnings. Tyrosine supplementation may help individuals maintain cognitive function in very hot, hypoxic, and/or cold conditions. However, more research is needed to clarify these and other postulated interventions.

## Introduction

Cognitive function defines performance in objective tasks that require conscious mental effort (Lamport et al., 2014). Such tasks include memory (verbal, spatial, and working), attention, and executive function (Lezak, 2004). These tasks are often categorized as either “simple” or “complex” (Ramsey and Kwon, 1992); simple tasks are those which require very simple perceptual motor skills (e.g., choice reaction time, memory recall), whereas complex tasks require a greater effort and/or attention, such as multiple/dual tasks [e.g., complex motor coordination, working memory tasks (Bradley and Higenbottam, 2003)]. However, categorization of cognitive tasks remains somewhat problematic as each task activates different regions of the brain (Qian et al., 2013) and individual familiarity with the task(s) may alter task classification. Based on previous literature (Gopinathan et al., 1988; Ramsey and Kwon, 1992; Bradley and Higenbottam, 2003; Hancock and Vasmatzidis, 2003; Gaoua, 2010) Table 1 provides a breakdown of which tasks are categorized as “simple” and “complex”—this review article will adhere to this classification. This categorization approach is recognized as simplistic and is utilized to aid readability of the presented Focused Review, with the caveat that readers should interpret this classification with care.

**Table 1:** **Categorization of simple and complex cognitive tasks**.

**Simple cognitive tasks**	**Complex cognitive tasks**
Mental transformation	Arithmetic efficiency
Monitoring	Attention
Memory recall	Complex motor coordination
Numerical vigilance	Dual tasks
Choice reaction time	Executive function
Short term memory	Mental addition
Simple arithmetic	Recall capacity
Simple visual orientation	Sustained attention
	Tracking
	Vigilance
	Visual motor tracking
	Working memory tasks

It is clear from previous work that different environmental conditions can negatively impact cognitive function. Past research has shown that hot (Hocking et al., 2001; Bandelow et al., 2010; Morley et al., 2012; Parker et al., 2013), cold (Marrao et al., 2005; Mäkinen et al., 2006; Adam et al., 2008a; Spitznagel et al., 2009; Muller et al., 2012; Taylor et al., 2014), and hypoxic (Kourtidou-Papadeli et al., 2008; de Aquino Lemos et al., 2012; Muller et al., 2012; Ando et al., 2013; Neuhaus and Hinkelbein, 2014) exposures can impair these cognitive processes in humans, and it appears that a combination of interrelated psycho-physiological pathways may be responsible for these deficits (as will be subsequently be reviewed). Moreover, we will discuss the pathways responsible for such cognitive alterations, relevant interventional strategies which may acquiesce these deficits, and finally recommendations for future research.

## Temperature and cognitive function

### Heat stress

Heat stress is recognized as an occupational hazard within the scientific literature, often attributed to compromised cognitive function (Ramsey et al., 1983; Tawatsupa et al., 2013). For example, when 17,000 safety observations were made over a 14 month period in factory workers, the relationship between the ambient temperature and unsafe behaviors formed a U-shaped curve. Specifically, minimum unsafe behaviors occurred when the ambient temperature was between 17 and 23°C, whereas temperatures outside of this range saw increasing unsafe behaviors displayed (Ramsey et al., 1983). Utilizing logistic regression (heat stress and occupational injury), it was demonstrated that ~20% of Taiwanese workers (*n* = 58.495) experienced occupational heat stress, which was strongly and significantly associated with workplace accident rates (Tawatsupa et al., 2013). The notion that indoor workers are generally sufficiently protected via air conditioning, fans, or other cooling systems does not apply to most industrial workplaces in low and middle income countries, that are located in hot regions of the world (Balakrishnan et al., 2010).

Occupations at risk of extreme heat exposure may include mining, shearing, farming, factory work, firefighting, and other emergency/military services (Arbury et al., 2014). Performing occupational tasks in close proximity to heat-generating equipment can also generate exceptionally hot environments. Additionally, several occupations (e.g., firefighting, chemical waste, and bomb disposal) require workers to wear impermeable protective clothing, which interferes with evaporative heat loss mechanisms and may overwhelm the ability of this primary heat loss effector system to maintain core temperature (*T*_*c*_) at ~37°C (Cheung et al., 2000; Armstrong et al., 2010).

Examining the relationship between heat stress and cognitive function is a challenging task as there are many confounding factors that may have influenced results from previous investigations. Examples of these methodological inconsistencies include severity of heat exposure (duration and temperature), the complexity of the cognitive task(s) completed, previous experience of participants, hydration status, the definition of heat stress (high ambient or *T*_*c*_), and the design employed to attain an increase in *T*_*c*_ (passive or exercise induced). Discussing each of these confounding issues in detail is beyond the scope of this focused review, however, the reader is directed to reviews which have addressed each of these issues at length (Hancock and Vasmatzidis, 2003; Hancock et al., 2007; Gaoua, 2010). When discussing both the mechanistic and observational evidence regarding heat stress induced cognitive alterations, the authors' have drawn upon data from studies using human passive heat stress models (to eliminate any confounding effect of exercise). Relevant recent literature in this area is collated within Table 2, and is drawn upon when making conclusions regarding the true effects of passive heat stress on cognitive function.

**Table 2:** **The effects of passive heat stress on cognitive function**.

**Author and date**	**Environment (temperature)**	**Duration**	**Cognitive assessment tool**	**Physiological response**	**Cognitive alterations**
Gaoua et al., 2012	50°C, 30% r.h.	15 min	Reaction time and task planning	Tc not elevated. Tsk elevated by ~3°C.	Decreased accuracy in task planning. Subjects took more time (41% increase) to find correct response.
Liu et al., 2013	50°C, 40% r.h.	45 min	Attention Network Test	Tc elevated to 38.5°C. Tsk not reported.	Impaired executive function.
Lenzuni et al., 2014	53.3–66.9°C	15–20 min	Driving in a straight line, and identifying a cue and reacting correctly	Tc elevated by ~0.3°C. Tsk not reported.	Impaired cognitive function in both simple and complex tasks ~50%
Berg et al., 2015	26°C	30 min	Peg transfer and intracoporeal knot tying	None reported	None despite reduced thermal comfort
Watkins et al., 2014	30°C, 50% r.h.	90 min with 15 min normothermic exposure at half-way	Dual task (tracking + simple reaction) and numerical vigilance	Tc elevated by ~0.2°C. Tsk elevated by ~4°C.	None
Gaoua et al., 2011	50°C, 50% r.h.	45 min	Attention and memory tests	Tc elevated to 38.6°C. Tsk elevated to 39.6°C.	Attention not impaired but memory impaired
Racinais et al., 2008	50°C, 50% r.h.	15 min walk at 3–5 km/h. Followed by 45 min passive exposure.	Attention, working and visual memory	Tc elevated to 38.8°C. Tsk elevated to 39°C.	Attention not impaired but working and visual memory impaired
Sun et al., 2012	50°C, 40% r.h.	60 min	Attention Network Test	Tc elevated to 38.4°C	Impaired executive function
Wijayanto et al., 2013	42°C	45 min	Short term memory	Delta Tc elevated by 0.31°C	None
Simmons et al., 2008	45°C, 50% r.h.	Until Tc increased by 1°C. (Time unknown)	Reaction time and numerical vigilance	Tc elevated by 1°C. Tsk elevated by 6°C.	Faster reaction time but reduced accuracy

It is generally accepted that simple task performance is less vulnerable to heat stress than complex task performance, a viewpoint that has been supported by recent literature reviews (Hancock, 1986; Pilcher et al., 2002; Hancock and Vasmatzidis, 2003; Gaoua, 2010). Complex tasks such as working memory (spatial span test, pattern recognition) were significantly impaired through heat stress [45 min at 50°C, 50% relative humidity (r.h.)], whereas simple attentional tasks (match to sample, choice reaction time, rapid visual information processing) were not affected (Gaoua et al., 2011). Moreover, Watkins et al. (2014) recently demonstrated that soccer goal line officials' ability to complete simple tasks (tracking, simple reaction time, and numerical vigilance) does not deteriorate during a 90 min passive exposure to 30°C, 40% r.h. However, the authors neglected to assess complex cognitive task performance and the severity of heat stress was unlikely to impose significant stress, especially using a passive model. Previous reviews also suggest that cognitive function is generally unaffected unless the external stimulus is sufficient in intensity and duration to increase *T*_*c*_ away from a homeostatic range approximate to 37°C (Hancock and Vasmatzidis, 2003). An early study led to this theory (Wilkinson et al., 1964), whereby as *T*_*c*_ increased to 38.5°C (through passive heating), simple task performance (vigilance) improved but complex task performance (mental addition) was compromised. However, in a later study, it was shown that passively heating individuals up to 39.05°C did not affect short or long term memory, verbal logic problems, and numerical subtraction performance (Holland et al., 1985). Therefore, it seems that in a hot environment *T*_*c*_ alone may not be a reliable predictor of cognitive performance decline. In support, recent research suggests that an increased skin temperature (*T*_*sk*_), independent of any rise in *T*_*c*_, may be responsible for any heat induced cognitive deteriorations (Gaoua et al., 2012). Participants in the aforementioned study were passively exposed to 50°C, 30% r.h. for ~15 min, and were required to complete simple (reaction time) and complex (working memory) tasks during the exposure. The results demonstrated that simple task performance was not affected, however complex task performance was significantly impaired. Thus, it appears that *T*_*sk*_ (which was significantly increased in the heat by ~3°C) and a reduced thermal comfort (~8 points on a 20 point scale) in the heat, whereby subjects reported more negative feelings (i.e., they felt hotter and less comfortable), were responsible for the reductions in complex task performance, which again were independent of any change in *T*_*c*_ (Gaoua et al., 2012). It may therefore be suggested that the subjective state of the individual could be a key factor affecting cognitive function in the heat, as these responses led to alterations in complex task performance independent of variations in *T*_*c*_. Indeed, selective head skin cooling (induced by three cooling packs) has been shown to preserve some complex cognitive functions (Gaoua et al., 2011). Therefore, it seems that increasing thermal comfort (rather than mitigating *T*_*c*_ increases) may be effective in maintaining complex cognitive function (and consequently safety) in passively experienced thermally stressful environments.

Recent investigations have utilized the **attention network** test (ANT) and **functional magnetic resonance imaging** (fMRI) to provide an insight into brain blood flow alterations upon exposure to heat stress, and how these changes affect aspects of the attention network (Jiang et al., 2013; Liu et al., 2013; Sun et al., 2013). The ANT (Macleod et al., 2010) is a tool used to measure the efficiency of three major attention networks; alerting (simple task; related to maintaining readiness), orienting (simple task; responsible for selecting the region of space or channel to be attended), and executive control (complex task; involved in resolving conflict among possible actions). These three aspects of attention differ from one another in brain activation locations (Petersen and Posner, 2012), hence, the application of fMRI has allowed researchers to accurately and simultaneously quantify how blood flow/activation in these areas vary upon exposure to environmental heat stress and relative to differential (simple or complex) cognitive tasks. In support of previous findings (see Table 2), passive heat exposure (1 h at 50°C, 40% r.h.) did not alter simple task (alerting and orienting) performance for reaction time or accuracy (Liu et al., 2013). The lack of heat induced cognitive alteration in these tasks appear to be due to increased activation in alternative brain regions i.e., a compensatory effect (see Table 3). Conversely, passive hyperthermia had a significant adverse effect on complex cognitive processes involving executive function, with reaction time increasing by ~22 ms. During the executive function task, there was no difference in activation at the anterior cingulate cortex (brain region involved in executive functioning) between the normothermic and hyperthermic groups, but again there appeared to be compensatory activation (Table 3). It is currently unclear why this type of complex task performance is consistently impaired by heat stress (Table 2) given the apparent compensatory activation. Similar results have been found elsewhere (Sun et al., 2012, 2013), however it is currently unknown if these changes were mediated by an increased *T*_*sk*_ (not reported) or *T*_*c*_ (peak ~38.5°C). A plausible explanation for these behavioral changes is that the increase in plasma serotonin (5-hydroxytryptamine; 5-HT) witnessed during passive heat stress (McMorris et al., 2006) inhibits the production of dopamine (DA), a neurotransmitter that appears to play a major role in complex task performance [executive function; (Rektor et al., 2003)]. As stated previously, the precise relationship between *T*_*sk*_ and 5-HT is currently unknown, therefore future work should examine if; (1) 5-HT plays a major role in these heat stress induced cognitive alterations, and (2) if plasma (human) and/or brain (animal models) 5-HT increases in response to an elevated *T*_*sk*_ and/or *T*_*c*_ differentially. To our knowledge, this relationship is yet to be examined.

KEY CONCEPT 1 **Attention Network**

Neuroscientific studies suggest that specific attentional functions are carried out by several interconnected brain networks. The attentional functions and related networks go under different names, but a classification into the three networks of alerting, orienting, and executive control is common. It is now understood that under many circumstances these networks interact and influence each other.

KEY CONCEPT 2 **Functional Magnetic Resonance Imaging**

This method highlights differences in brain activity by measuring related blood oxygenation levels. It yields information regarding relative differences in brain activity when comparing two or more experimental conditions and thus offers useful insight as to which brain areas are selectively active during certain mental processes.

**Table 3:** **Summary of activated and depressed brain regions during passive heat stress (Liu et al., 2013)**.

	**Alerting network**	**Orienting network**	**Executive network**
Increased activity	Right superior frontal gyrus	Temporal lobe	Frontal lobe Superior temporal gyrus Lingual gyrus
Depressed activity	Right middle occipital gyrus	Frontal Parietal lobe	Post-central gyrus
	Left inferior parietal lobe	Occipital lobe	
	Left culmen		

The available evidence demonstrates that heat stress related cognitive decline is primarily mediated by a reduction in thermal comfort (Gaoua et al., 2012) and/or changes in regional brain blood flow (Liu et al., 2013; Qian et al., 2013). Although the involvement of 5-HT is not yet well established, evidence has shown that augmenting the bioavailability of the amino acid tyrosine (a precursor for DA synthesis) may preserve cognitive function during thermal stress (Wurtman et al., 1980). In support, it has recently been shown that tyrosine (6.5 g) ingested 90 min prior to passive heat stress (90 min at 45°C, 30% r.h.) significantly decreased event related potential (P300) latency and contingent negative variation latency compared with a placebo (Kishore et al., 2013). Moreover, there was a significant increase in plasma DA concentrations when participants ingested **tyrosine** (Kishore et al., 2013), an effect which may be responsible for the improved cognitive function. Conversely, it has been shown that 150 mg·kg^−1^ tyrosine ingestion prior to exhaustive exercise did not affect cognitive function in a warm environment compared to a placebo (Watson et al., 2012). However, the environmental conditions (i.e., thermal stress) may not have been sufficient in magnitude to increase plasma 5-HT, as to our knowledge, this has not been shown to increase in such a mild environment. Thus, as plasma DA levels were not assessed in the aforementioned study, and tyrosine works by increasing DA concentrations, it is impossible to draw conclusions about the true effects of tyrosine in this work. Although there are very few well controlled studies in this area, the evidence available (Kishore et al., 2013) suggests that ingestion of tyrosine may be an effective strategy to maintain cognitive function during passive heat stress. Finally, as sensory displeasure (decreased thermal comfort elicited through an increased *T*_*sk*_) may be the primary factor mediating heat stress induced cognitive disturbances (Gaoua et al., 2012), it is reasonable to suggest that improving thermal comfort (through an acute reduction in *T*_*sk*_) may also combat the negative side effects of heat stress on complex cognitive functioning. However, results to date have not wholly supported this notion. For example, a recent study has shown that ingestion of menthol lozenges had no such effect (Zhang et al., 2014) during simulated firefighting in the heat. However, when applied directly to the skin, menthol has a stimulating action on the peripheral cold receptor TRPM8 (Eccles, 2000), thus, the ingestion of menthol lozenges is likely to be an inferior intervention compared to direct menthol application to the skin. It is important to note that the effect of menthol on thermal comfort is dose dependent, whereby concentrations of <2% elicit a cool sensation (Cliff and Green, 1994), but concentrations >2% may cause irritation and burning sensations (Yosipovitch et al., 1996). Research is needed to determine if topically applied menthol (at concentrations <2%) is effective at improving thermal comfort and consequently maintaining complex cognitive function during heat stress.

KEY CONCEPT 3 **Tyrosine**

Tyrosine, a non-essential amino acid synthesized in the liver from phenylalanine, is a precursor for the synthesis of catecholamines. Nutritional supplementation of tyrosine increases its ratio to other large chain amino acids, and can result in a greater cerebral uptake of dopamine and noradrenaline. Evidence suggests that this response helps maintain cognitive function in extreme environmental conditions.

### Cold stress

Cold stress is experienced in occupational (military, fishing trawlers, emergency disaster workers) and athletic (winter sports) settings (Muller et al., 2012). It appears that both moderate and extreme reductions in ambient temperature may have a negative effect on cognitive function (Banderet et al., 1986; Palinkas, 2001). Specifically, cold exposure (−20 to 10°C) has led to decrements in memory [complex task (Thomas et al., 1989; Patil et al., 1995)], vigilance [complex task (Flouris et al., 2007)], reaction time [simple task (Teichner, 1958; Ellis, 1982)], and decision making [complex task; see Table 4; (Watkins et al., 2014)]. Such consistent findings across such diverse ambient temperatures may be explained by traditional theories of cold induced cognitive decrement (Teichner, 1958; Enander, 1987; Muller et al., 2012). The distraction theory (Teichner, 1958) explains that exposure to cold conditions provides alternative stimuli to interrupt focus which would otherwise be fixed on the cognitive task at hand (i.e., attention is focused on feeling cold rather than completing the cognitive task provided). This theory is supported by recent findings where temperatures as mild as 10°C (Muller et al., 2012) may have provided enough of a sensory challenge to distract participants from a set cognitive task. Regression analysis from a recent study (Watkins et al., 2014) reported a significant relationship between alterations in thermal comfort and cognitive function in the cold.

**Table 4:** **The effects of passive cold exposure on cognitive function**.

**Author and date**	**Environment (temperature)**	**Duration**	**Cognitive assessment tool**	**Physiological response**	**Cognitive alterations**
Shurtleff et al., 1994	4°C	30 min	Match to Sample	Increased systolic blood pressure following cold exposure	Cold exposure reduced matching accuracy
Patil et al., 1995	2–3°C (Cold Water Immersion)	3 min	Variety of simple and complex tasks	Increased systolic and diastolic blood pressure following cold water immersion	Cold exposure increased alertness, but worsened short-term memory
Banderet et al., 1986	Night: −4 to −10°C; Day: −23 to −25°C	5 days	Pattern and number comparison, grammatical reasoning, coding	None reported	All cognitive tasks impaired aside from grammatical reasoning
Marrao et al., 2005	9 day range: −24 to 4.4°C	9 days	Logical planning, reasoning, vigilance	No significant thermoregulatory changes	None
Mäkinen et al., 2006	10°C	10 days	Cognitive Battery (ANAM-ICE)	Significant reductions in Tc, Tsk and finger temperature across exposure	Cold exposure increased response time, decreased accuracy, and efficiency of tasks
O'Brien et al., 2007	10 or 15°C (Cold Water Immersion). Subsequent cold air exposure until Tcore reached 35.5°C.	N/A	Match to Sample, complex reaction time, logical reasoning, visual vigilance, addition and subtraction, repeated acquisition	Cold water immersion reduced Tc by 0.3 to 1°C. Tsk reduced to ~26°C. Finger temperature reduced to ~15°C.	Cognitive function was not affected by cold water immersion

*Regard et al., 1989; Racinais et al., 2008; Berg et al., 2015*.

Exposure to cold conditions alters the concentration of central catecholamines [DA, epinephrine and norepinephrine (Avakian et al., 1984)]. Alterations in levels of central catecholamines (Rauch and Lieberman, 1990) may have a detrimental effect on cognition as brain regions such as the prefrontal cortex are reliant on these neurotransmitters for normal function (Rektor et al., 2003; Friston et al., 2014). There is a plethora of evidence which demonstrates that tyrosine supplementation improves cognitive function during acute cold stress (Shurtleff et al., 1993, 1994; Yeghiayan et al., 2001; Palinkas et al., 2005; Mahoney et al., 2007; O'Brien et al., 2007). Given that tyrosine is a precursor for the synthesis of norepinephrine and DA, these studies support the notion that alterations in catecholamine concentrations may play a role for cold stress induced cognitive impairment. However, further support is needed to clarify if this is the case in humans.

Tyrosine supplementation is likely to improve cognitive function during exposure to cold environmental conditions (as previously described). Similar augmentation of cognitive function was observed following the use of caffeine, although this was during exposure to multiple stressors [cold, intense physical and psychological stress (Lieberman et al., 2002)]. The implementation of multiple stressors makes it difficult to attribute any improvements in cognitive function to caffeine supplementation. However, as caffeine may increase metabolic rate (and potentially increase in *T*_*c*_; Poehlman et al., 1985) this may provide a favorable physiological effect when exposed to cold conditions. Adequate clothing for cold, dry environments should aim to block airflow but enable water vapor to dissipate if sweating occurs, i.e., to maintain body heat balance (Holmér, 1988). Cold acclimation or acclimatization is suggested to result in reduced vasoconstriction, increased skin temperature, delayed onset of shivering, dampened release of stress hormones, and reduced thermal discomfort (Mäkinen et al., 2006). Specifically, a reduction in cold stress from acclimation or acclimatization should in theory limit shivering and thermal discomfort and thus potentially limit the level of distraction and thus may positively influence cognitive function. Despite this, it was demonstrated that 10 days repeated exposure to 10°C did not significantly alter cognitive function, including accuracy, efficiency and response time, when compared to control (Mäkinen et al., 2006).

Given the equivocal nature of acclimation/acclimatization and tyrosine supplementation on cold induced cognitive function disturbances, appropriate clothing to maintain thermal comfort is the only robust intervention presently available. Evidently, further interventional work is required to positively influence the cold environment cognition nexus.

## Hypoxia and cognitive function

Hypoxia is defined as a reduction in alveolar oxygen partial pressure [PaO_2_ (Petrassi et al., 2012)]. Reduced PaO_2_ availability is experienced at high altitudes (~1500–7500 m) and has been shown to have detrimental effects on cognitive function in human subjects (Abraini et al., 1998; Adam et al., 2008b; de Aquino Lemos et al., 2012). In accordance with the previous sections, there are a number of confounding variables that must be considered when reviewing the relationship between hypoxia and cognitive function. These include altitude severity, ambient temperature, the addition of exercise, variability in physiological responses, and the differences in barometric pressure (Virués-Ortega et al., 2004). The present section will primarily review studies which assess changes in cognitive function in a laboratory setting, and studies which have used passive hypoxic exposures (normobaric and hypobaric).

It is generally accepted that there is a negative correlation between altitude and cognitive function (Table 5; Li et al., 2000; Pickard, 2002; Rainford and Gradwell, 2006; Merz et al., 2013; Neuhaus and Hinkelbein, 2014; Xu et al., 2014). Increases in reaction time (simple task) have been observed at altitudes exceeding 5000 m, an effect which persisted 75 days after participants returned to sea level following acute altitude exposure (Cavaletti and Tredici, 1993). As individuals ascend to altitude >5000 m, decrements in all facets of memory (complex tasks) have been observed; including learning (Bouquet et al., 1999), spatial (Nelson, 1982), and working memory (Yan et al., 2011; Champod et al., 2013; Malle et al., 2013). More recently, 90 min exposure to a simulated altitude of 6096 m [10% oxygen (O_2_)] significantly deteriorated many aspects of cognitive function, including complex attention, executive function, and cognitive flexibility (Turner et al., 2015). In the field, Kramer et al. (1993) demonstrated using a battery of cognitive tests (pattern comparison, code substitution, reaction time, memory) that altitude induced deficits in short term memory and reaction time. The tests took place at Genet Basin, 14,200 ft (4328 m) above sea level, and results were compared with a matched control group who completed the same tests at sea level. Interestingly, the climber's ability to learn new skills was also monitored upon return to sea level, and it was shown to be impaired for up to 2 weeks. These results support the notion that high altitude exposure causes acute and chronic deficits in cognitive function.

**Table 5:** **The effects of passive hypoxia on cognitive function**.

**Author and date**	**Environment (altitude)**	**Duration**	**Cognitive assessment tool**	**Physiological response**	**Cognitive alterations**
Adam et al., 2008b	4300 m	5 days	Mental addition	None reported	Cognitive performance was better at sea level than altitude
de Aquino Lemos et al., 2012	4500 m	24 h	Vigor, attention, visual and working memory, concentration, executive functions, inhibitory control, and speed of mental processing	Decreased sleep time and rapid eye movement sleep	Decreased performance in all facets of cognitive function measured
Merz et al., 2013	4497, 5533, 6265, 6865, 7546 m	N/A	Saccadic Eye Movement, Neuropsychological Testing	Significant reduction in SaO_2_ as ascent increases	Neither parameters effect by altitude exposure
Kourtidou-Papadeli et al., 2008	8000 ft	16 min	Multi attribute task batter (MATB)	None reported	Increase in errors and a decrease in tracking performance
Pavlicek et al., 2005	3 profiles: 450-1500–3000 (1); 450–1500–4500 (2); 450–650–650 (control)	30 min at each simulated altitude	Word fluency, three word association task. Tachistoscopic lexical decision task	None reported	No change in performance between groups.
Shukitt et al., 1988	Exposure to: 21, 17, 21, 13, and 21% oxygen	3 days at each altitude	Addition, coding, computer interaction, map/compass, number comparison, pattern comparison, pattern recognition	Significant reduction in SaO_2_ at 13% only	Cognition and mood were only effected on the first day at 13%
Tripathi et al., 2005	10,500 ft	6 days	Working Memory and Vigilance Tasks	Symptoms of acute altitude sickness	No differences in cognitive performance across days
Yan et al., 2011	High landers: 2616–4200 m; lowlanders < 400 m	High landers (≥18 years exposure).	Verbal working memory	High landers had decreased activation in numerous brain regions compared to low landers	Longer reaction time and decreased accuracy in highlanders
Li et al., 2000	2800, 3600, and 4400 m	60 min at each altitude	Simple reaction time and four choice reaction time.	None reported	Four choice reaction time performance decreases at 3600 and 4400 m
Abraini et al., 1998	Ascent to 8848 m (Mt. Everest simulation)	31 days	Visual reaction time, psychomotor ability and number ordination	Significant reduction in SaO_2_ as a function of elevation	Cognitive decrements at ≥8000 m and decrements up to 3 days after return to sea level
Hewett et al., 2009	Sea level, 8000, 10,000, 12,000, 14,000 ft	45 min at each altitude	Cognitive Battery (Cogscreen—HE)	Significant reduction in SaO_2_ as a function of elevation	No differences in cognitive performance across altitudes
Gao et al., 2014	Lived at 4500 m for 1–5 years compared to a sea level control	N/A	Cognitive Battery (WHO neurobehavioral core test battery) and Raven standard progressive matrices	Highlanders exhibited a significant reduction in basal SaO_2_ and BDNF, and an increased serum S100B compared to sea level controls	Highlanders exhibited a poorer cognitive function across a range of simple and complex tasks. Mood state was also adversely affected
Nelson, 1982	Sea level, 3810; 5000 m.	35 day expedition	Bender visual motor Gestalt test and Porteus maze test	None reported	Marked deterioration in cognitive functioning at 5000 m. Mood state was also severly affected
Kobrick and Appleton, 1971	15,000 ft	48 h	Near and far visual acuity, steropsis, binocular depth perception, critical flicker fusion, dark adaptation, response time to peripheral signals	None reported	Decrements observed in all visual tasks which peaked after 60 min of exposure. Task performance gradually recovered throughout the exposure.
Regard et al., 1989	Climbers had previously reached 8500 m without supplementary oxygen	N/A	Concentration, short term memory, ability to shift concepts	Malfunctioning of the bifronto-temporo-limbic structures	Majority of climbers sampled suffered impaired concentration, memory, and ability to control errors after returning to sea level
Kramer et al., 1993	14,200 ft (5–9 day ascent)	18–26 days	Pattern comparison, choice reaction time, memory, tapping, code substitution	Presence of moderate acute mountain sickness in all subjects	Climbers showed deficits in learning and retention in memory tasks and slower reaction time.

An fMRI study conducted at sea level revealed that subjects that had experienced chronic hypobaric hypoxia (>18 years; ~2616–4200 m) displayed decreased activation in a number of brain regions (various gyri, pyramis of vermis, and thalamus) as well as reduced performance during complex cognitive tasks (working memory) when compared to their sea level counterparts (Yan et al., 2011). Interestingly, another study revealed that those exposed to chronic (7 months) moderate hypobaric hypoxia (2260 m) performed similarly to a group of sea level residents during a battery of complex tasks taken place at sea level (verbal, spatial, long term memory), although impairments in simple task performance were evident [short term memory (Zhang et al., 2011)]. Finally, acute ascents to high altitudes can cause alterations to cerebral architecture (Wilson et al., 2009). The use of fMRI revealed that world-class climbers (sea level residents) acutely exposed to hypobaric hypoxia displayed modifications to cerebral tissue (Paola et al., 2008), in comparison to a sea level control group. Specifically, the climbers displayed a reduction in left angular gyrus volume, a key brain region involved in movement control and planning (Paola et al., 2008).

Traditionally it has been thought that acclimatization to various environmental conditions could be used as an intervention to improve cognitive function. Although this may be true with regard to athletic performance, fMRI has uncovered a number of negative adaptations in those chronically exposed to hypobaric hypoxia [~2600–6500 m (Paola et al., 2008; Zhang et al., 2010; Yan et al., 2011)]. For example, chronic exposure to hypobaric hypoxia has been shown to reduce the cerebral volume of both gray and white matter [>15 years at altitude (Zhang et al., 2010)]. In highland dwellers, gray matter volume was reduced in the prefrontal cortex, the anterior insular cortex, the anterior cingulate cortex, and the lingual cortex (Zhang et al., 2010), and these changes were still visible 1 year after returning to sea level. Alternatively, the cognitive deficits suffered during chronic high altitude exposure may be due to **hyperhomocysteinemia**. Elevated circulating levels of homocysteine have been associated with declines in cognitive function in several geriatric population based studies (Wright et al., 2004), and there is a strong correlation between homocysteine in plasma and cognitive impairment during chronic (18 months) hypoxia exposure (Sharma et al., 2014). Interestingly, this study further supports the notion that altitude acclimatization does not induce a favorable adaptation with regards to cognitive function.

KEY CONCEPT 4 **Hyperhomocysteinemia**

Hyperhomocysteinaemia is a condition characterized by an abnormally high level of homocysteine present in the circulation. Interestingly, a recent study found that cognitive impairment (visuo-spatial executive, attention, delayed recall, and procedural memory related cognitive domains) is progressively associated with duration of stay at high altitude and is strongly correlated with elevated homocysteine in plasma.

The mechanisms which explain how acute hypoxia impairs cognitive function are not completely clear, although it is likely an amalgamation of factors which may include neuronal damage (Bjursten et al., 2010), the onset of altitude sickness (Wilson et al., 2009), and fatigue (Virués-Ortega et al., 2004). Off-setting this response may be achievable through the administration of Dehydroepiandrosterone (DHEA), which has been reported to curtail neuronal damage [measured *in vivo* via plasma S100β quantification (Bjursten et al., 2010; Winter et al., 2014)] through enhancing the production of brain derived neurotrophic factor (Rahmani et al., 2013; Sakr et al., 2014). Furthermore, DHEA administration has been shown to improve complex cognitive functioning and increase levels of central catecholamines in the rat (Sakr et al., 2014). This is significant as the maintenance of dopamine is necessary for executive functioning (Rektor et al., 2003). Future research may consider the implementation of DHEA for its neuro-protective effects, particularly in those individuals exposed to high altitudes on a regular basis or for prolonged periods. It is important to stress that DHEA is on the World Anti-Doping Agency (WADA) banned substances list and thus is prohibited during athletic competition (although this would not preclude its acute short-term use in recreational or occupational settings). Other supplementary aids, which are not banned by WADA, such as tyrosine, have been used successfully to offset hypoxia-induced symptoms, adverse moods, and cognitive decrement (Banderet and Lieberman, 1989). Additionally, creatine supplementation significantly maintained cognitive function in a variety of tasks compared with placebo supplementation during severe hypoxia [90 min at 6096 m (Turner et al., 2015)]. Supplementary O_2_ appears to be a logical aid which may offset the negative side effects of hypobaric hypoxia on cognitive function, although a distinct lack of literature exists on this topic. Interestingly, altitude induced reductions in endogenous O_2_ saturation have not been significantly correlated with cognitive decrement, suggesting inter and intra individual variation regarding the cognition hypoxia nexus (Gao et al., 2014). Further research is required to determine if these ergogenic aids improve cognitive function at high altitude.

## Future research directions

Further research is required to reach a more definitive conclusion regarding the effects of passive exposure to extreme environments on cognitive function in humans, and to expand the knowledge and understanding of the potential intervention techniques that can be employed. These advancements are necessary to improve cognitive function and safety during exposure to such environments in a variety of contexts.

As stated, ANT and fMRI have provided an insight into brain blood flow alterations and how the attention network is affected by changes in environmental conditions (Liu et al., 2013; Sun et al., 2013). Such an understanding has been disproportionally advanced in the heat in comparison to cold and hypoxic environments, with intuitive and novel use of ANT alongside fMRI (Jiang et al., 2013; Liu et al., 2013; Sun et al., 2013). Such approaches are required for the remaining environmental extremes. The use of biochemical measures (particularly catecholamines) will increase mechanistic cause and effect evidence relative to the above outlined paradigms, and may aid the development of additional intervention techniques to reduce environmentally mediated cognitive decrements.

There also appears to be a lack of reliability data in the majority of studies utilized within this review. Reliability statistics, including the change in mean, coefficient of variation, intraclass correlation coefficient, and typical error for these cognitive tests (and other experimental procedures) should be overtly reported, as to ensure that the true experimental effects can be correctly disseminated, and to demonstrate the reproducibility of the cognitive tests used. There also appears to be heterogeneity among dependent measures (i.e., cognitive tests) used between studies, making them difficult to compare and thus draw meaningful conclusions. It is recommended that future researchers utilize the ANT in such studies, as this test is simple to implement, and can directly measure the efficiency of the three major attention networks (alerting, orienting, and executive function). Moreover, rigorous familiarization for the cognitive tests (and other experimental procedures) utilized will reduce learning effects which were likely evident in at least some of the previous work outlined within this review; increasing the reliability that the change in cognitive function is caused by environmental exposure and not poor experimental design. For example, experienced workers appear to be less vulnerable to the effects of heat stress than inexperienced workers because of task familiarity (Hancock, 1982). Thus, added experience of the task allows it to become more autonomous and “attention free” (Nunneley et al., 1979). This presents a major confounding factor in previous studies, which is why researchers should implement a minimum of one familiarization session before experimental trials are conducted. The presence of a learning effect can be ruled out when two or more familiarization tests are shown to be highly reliable [normally via intraclass correlations and coefficient of variation analysis (McGawley and Bishop, 2006)]. Furthermore, the proposed interventions could likely induce psychosomatic (i.e., you consume an ice based drink and you believe your body temperature will reduce, whether such a reduction actually occurs or not) and/or *bona fide* physiological responses (i.e., exogenous oxygenation administration increasing various tissue oxygenation indexes whilst within a hypoxic environment), future research should explore these subtleties (as they are currently not clear) to optimize interventional techniques and provide further mechanistic evidence relative to the content of this Focused Review.

If adhered to, these guidelines could lead to greater knowledge and understanding of the cognitive decrement that often occurs during passive exposure to extreme environments. In turn, this may lead to the development of further interventional techniques, and thus ultimately enable practitioners to reduce the decline in cognitive function during extreme environmental exposure. These outcomes could prove life-saving when applied to many occupational settings.

## Conclusion

This review has highlighted the detrimental effects of passive exposure to environmental extremes of heat, cold, and hypoxia on cognitive function in simple and complex tasks. A decline in cognitive function can be attributed to a number of mechanisms, dependent on the environment to which the individual is exposed. It should be noted that such declines, across all the environmental conditions, will have some aspect of inter and intra-individual variation.

Alterations in blood flow and sensory displeasure (Gaoua, 2010; Gaoua et al., 2012; Jiang et al., 2013; Sun et al., 2013), hyperhomocysteinemia and potential neuronal damage (Rothermundt et al., 2003; Bjursten et al., 2010; Koh and Lee, 2014; Sharma et al., 2014), and a decrease in catecholamine availability (Shurtleff et al., 1994; Starcke and Brand, 2012) combined with psychological factors (Teichner, 1958; Enander, 1987), appear to be responsible for reductions in cognitive function during hot, hypoxic, and cold exposure, respectively. Although mechanisms detailed within this review have outlined how environmentally mediated changes in cognitive function may occur, there is still a need to unequivocally determine mechanistic cause and effect data to understand the psycho-physiological mechanisms underpinning these cognitive changes during exposure to such environments.

A variety of interventional techniques can be implemented to potentially combat the negative effects of exposure across the aforementioned paradigms. The use of cooling interventions (Nunneley et al., 1982; Bandelow et al., 2010; Lee et al., 2014) in the heat, clothing and exercise (Doubt, 1991; Gavin, 2003) in the cold, and nutritional interventions (Banderet and Lieberman, 1989; Lieberman et al., 2002; Baker, 2013; Meeusen, 2014; Coull et al., 2015) across all three extreme environments are supported with evidence suggesting that they are often successful at maintaining cognitive function. Further well controlled research should also investigate if tyrosine supplementation is effective in maintaining cognitive function during exposure to heat, hypoxia, or cold environments. Future studies should focus on implementing empirically informed techniques, and should aim to discover additional methods by which optimal cognitive function can be maintained in extreme environments, in order to avoid detrimental outcomes.

### Conflict of interest statement

The authors declare that the research was conducted in the absence of any commercial or financial relationships that could be construed as a potential conflict of interest.

## Abbreviations

°C: Degrees Celsius
5-HT: 5-hydroxytryptamine
ANT: Attention Network Test
DA: Dopamine
DHEA: Dehydroepiandrosterone
fMRI: Functional Magnetic Resonance Imaging
m: Meter
min: Minutes
ms: Millisecond
O_2_: Oxygen
PaO_2_: Partial Pressure of Oxygen
r.h.: Relative Humidity
*T*_*C*_: Core Temperature
TRPM8: Transient Receptor Potential Melastatin 8
*T*_*SK*_: Skin Temperature
WADA: World Anti-Doping Agency.
